# Creation and validation of the 4-item BriefPCS-chronic through methodological triangulation

**DOI:** 10.1186/s12955-020-01346-8

**Published:** 2020-05-07

**Authors:** David M. Walton, Swati Mehta, Wonjin Seo, Joy C. MacDermid

**Affiliations:** 1 Department of Health and Rehabilitation Sciences, Western University, London, Ontario Canada; 2 School of Physical Therapy, Western University, Rm EC1443, 1201 Western Rd, London, Ontario N6G 1H1 Canada

**Keywords:** Pain catastrophizing, Outcome Measures, Rasch analysis, Confirmatory factor analysis, Content analysis, Methodological triangulation

## Abstract

**Background:**

The Pain Catastrophizing Scale (PCS) is a widely used self-report tool to evaluate pain related catastrophizing. The PCS was developed using classical test theory and has been shown to be psychometrically sound among various populations. However, it’s current three subscales are rarely used in clinical practice, offering potential for an abbreviated version that reduces administrative burden and can be used to estimate full scale scores, yet is not bound by the inclusion of items from each subscale. Hence, the aim of the current study was to develop a unidimensional abbreviated version of the PCS through findings from qualitative, classical test theory, and newer Rasch analysis.

**Methods:**

The current cross-sectional study used data from the Quebec Pain Registry (*n* = 5646) to obtain PCS scores of people seeking care at tertiary chronic pain centres. To develop an abbreviated unidimensional tool, items were removed based on triangulation of qualitative review of each item and response, corrected item-total correlations, and Rasch analysis. Confirmatory factor analysis was conducted on the final remaining items to confirm the tool was assessing a single latent construct (catastrophizing). Fit was assessed using the cumulative fit index (CFI), Tucker Lewis Index (TLI), and root-mean-squared error of approximation (RMSEA).

**Results:**

After triangulation, a final abbreviated 4-item scale showed adequate model fit with a strong correlation (*r* > 0.95) with the original scale and properties that were stable across age, sex, cause, and medicolegal status. Additionally, the brief version addressed some problematic wording on some items on the original scale. Both the original and new abbreviated tool were associated with the Beck Depression Inventory and the Brief Pain Inventory at the same magnitude.

**Conclusion:**

The abbreviated scale may allow for a decrease in administrator burden and greater clinical uptake when a quick screen for exaggerated negative orientation towards pain is needed.

## Introduction

Pain-related catastrophizing, defined as an exaggerated negative orientation towards pain [1], has become a widely recognized construct for explaining significant variance in experimental [2, 3] and clinical [4, 5] pain conditions. Pain-related catastrophizing has also demonstrated consistent prognostic association with outcomes following motor vehicle collision [6], surgery [7, 8] and conservative rehabilitation [9]. The Pain Catastrophizing Scale (PCS [3]) is a widely used tool for quantifying pain-related catastrophizing. It consists of 13 items related to the experience of pain including ‘I feel I can’t stand it anymore’ and ‘I can’t seem to keep it out of my mind’. Prior authors have used exploratory [10], confirmatory [11, 12] and Rasch-based [13] approaches to identify 3 sub-factors within the PCS: rumination, helplessness/hopelessness, and magnification. Despite this, the subscale scores are rarely reported separately in clinical research or practice, raising the possibility that the subscales are not always necessary.

If a single summative scale score is preferred, then 13 items may be unnecessary for many routine clinical encounters. Prior authors have reported high internal consistency of the original PCS (α ≥ 0.95) [12, 14] that suggests a degree of item redundancy and the potential for an abbreviated scale [15]. Bot et al. [1], McWilliams et al. [16] and Darnall et al. [17] have all previously published shortened (4-item, 6-item, and 3-item, respectively) versions of the PCS. Both Bot et al. [18] and McWilliam et al. [16] have used Classical Test Theory methods in samples of upper extremity or mixed chronic pain to derive their shortened versions and intentionally included items drawn from each of the 3 PCS sub-domains. McWilliams et al. [16] found that the 4-item version endorsed by Bot et al. did not satisfy all a priori criteria for adequate measurement properties, favoring the 6-item version instead. Neither has yet been tested for invariance across clinically relevant subgroups like sex, age, or symptom duration. Darnall et al. [17] employed more qualitative cognitive interviews to adapt and refine the scale contents and instructions for use as a brief tool for daily administration. Employing three approaches, they derived a new 3-item ‘daily PCS’ that included one item from each of the 3 original subscales.

To our knowledge the prior efforts at abbreviation have yet to see widespread adoption. We believe that, in the absence of clear rationale for retaining the 3-factor structure in most clinical encounters, a better priority would be to create an abbreviated version that can be easily used to predict full-scale scores rather than adhere to a 3-factor structure. Newer measurement approaches including Rasch modeling for ordinal scales [8] offers the potential to conduct deeper exploration of individual item function, and large databases allow scale evaluation from different statistical and theoretical perspectives in the same population. Qualitative or theoretical review is rarely prioritized over statistical methods for scale interpretation, though we suggest that a triangulation of approaches between classical and newer statistical methods and theoretical review will lead to even better scale development. To that end the purpose of this study was to triangulate findings across qualitative, Classical Test Theory, and Rasch-based analyses to arrive at a psychometrically sound, unidimensional, abbreviated version of the PCS that could be confidently applied across conditions and clinical subgroups, reducing burden while providing scores that are comparable to those of the original version.

## Methods

The database for this analysis consisted of 5646 PCS scores obtained through the Quebec Pain Registry (QPR) for chronic pain problems. The QPR is a province-wide administrative and research database that provides standardized data on a large cohort of patients with chronic pain referred to tertiary care pain clinics. Participant phenotypes are described using a set of common demographic and clinical measures based on uniform and validated tools [19]. It has undergone considerable data fidelity checking with each dataset checked by two independent research nurses. Data were provided for participants between October 2008 and December 2014. Inclusion criteria included adult (18 years old and above) community-dwelling males and females with non-cancer pain that could read and understand conversational French or English. Participants completed the full PCS as originally described by Sullivan [3], the Brief Pain Inventory Interference subscale [20] that provided two scores: Physical Interference and Affective Interference [21], and the Beck Depression Inventory – II, a well-supported measure of depressive symptoms that has been used extensively in pain studies [22]. The database also included longitudinal follow-up data, but only the baseline data were used in the current study. Raw score responses to each individual item on the PCS and all other scales were extracted from the QPR data to form the study database for the current analysis. Ethical approval was obtained from the relevant institutions prior to data collection. No additional ethical approval was sought for this secondary analysis of de-identified data.

## Analyses

### Qualitative / conceptual review

A detailed, line-by-line qualitative interpretation of each item and response option was reviewed by two authors who were not involved with the original design of the tool (DW, SM) to provide independent perspectives on the fit of the items with routine clinical practice and the conceptual basis of catastrophizing. Discrepancies in perspectives were resolved through discussion. Items were flagged at this point for reasons of possible mismatch between conceptual theory and wording. These reasons included: being unable to form a meaningful sentence through combination of item (stem) and response options, items that contained multiple concepts (e.g. were ‘double barrelled’), or apparent redundancies. The content of each item was also mapped to the original theoretical framework of catastrophizing as an exaggerated or irrational appraisal of pain, as described by Sullivan [3]. No items were removed at this stage, rather the results of the conceptual analysis were considered alongside the quantitative analyses to arrive at final decisions regarding retention/removal.

### Corrected item-total correlations

Corrected item-total correlations (ITCs) were evaluated through creation of an intercorrelation matrix where each item served as the independent variable and the summed score from all remaining items was the dependent variable. A hypothesis to drive the analysis was that all items should individually be significantly correlated with the summed score from all other items, aligning with our priority of creating a brief tool that would correlate well with the original full version. Out of respect for the well-established value of the PCS we introduced methods to avoid spurious findings or premature removal/retention of an item through use of three independent random samples drawn from the database. Each independent sample comprised *n* = 130 responses (10 subjects per item). An a priori requirement of a minimum corrected ITC of *r* = 0.70 for each item *across all 3 independent samples* was set as a threshold for retention, thereby reducing the likelihood of removing an item by chance. Prior to removal the items were compared against the qualitative interpretation in step 1 and only those that were flagged in both analyses (clearly problematic) were removed at this stage as a first pass scale shortening.

### Rasch analysis

Rasch analysis using the remaining items was conducted in accordance with standard practice using RUMM2030 (RUMMLab, Australia) software. Rasch is a probabilistic modeling approach that assumes location of each respondent (termed ‘persons’) on the continuum of the latent construct can be predicted by virtue of knowing that person’s responses to each item, and that response to each item can be predicted by knowing that person’s location on the construct [23]. Any deviations between predicted and observed scores are termed ‘misfits’ and can be further explored through detailed analysis. Rasch analysis allows exploration down to the level of each item/response combination, and provides evidence to support appropriate ordering of response options (termed ‘thresholds’), targeting of each person to the scale, unidimensionality, and differential item functioning (DIF). We followed the general approach of Pallant and colleagues [24] using the partial credit model. Model fit was primarily evaluated through a corrected chi square test in which predicted and observed scores were evaluated for agreement beyond chance. Where significant misfit was identified detailed exploration was conducted. Response thresholds were explored for proper ordering using probability curves. DIF was explored to determine the effects on predictive accuracy influenced by the following variables: sex (male/female), cause (traumatic/insidious), medicolegal status (involved/not involved), and age (< 55 years/55 years or older) through an ANOVA-based approach that stratifies the sample based on level of DIF variable, then compares the residuals between levels of stratification to identify potential differences in scale function. Unidimensionality was explored by creating two separate scales based on factor analysis of residual (error) terms, then comparing predicted location on the two subscales using paired t-tests. If the difference in predicted person locations was not different between the two subscales, adequate unidimensionality was assumed. This is identical to approaches we have used previously for Rasch analysis of the PCS [13], and readers are directed to the prior work for more detailed description of the analysis. Where problems were identified, the research team considered the best approach to each based on the nature of the problem and the goal of the study. If modifications were made the scale was retested using the existing data and a separate independent cohort drawn from the larger database. Upon arriving at an appropriate scale the person separation index (PSI) was calculated as an indicator of internal consistency, with thresholds of PSI ≥ 0.80 considered adequate for person-level comparisons and PSI ≥ 0.70 adequate for group-level comparisons [23]. Sample size for Rasch analysis is not easily estimable, with published manuscripts describing samples of 100 to 1000 participants. As the primary statistic (chi square) is highly sensitive to sample size, two random draws of 250 responses each (*n* = 500 total) were extracted from the database, safely above the minimum needed to avoid random bias while below a level at which a significant chi square would be difficult to avoid.

### Confirmatory factor analysis

As a second step to validate unidimensionality from another perspective, confirmatory factor analysis (CFA) was conducted from a Classical Test Theory-based perspective using MPlus v6.2 software (Muthen & Muthen Inc.). The goal was to arrive at a set of items that loaded adequately on a single latent construct, but two- or three-factor solutions were considered (where appropriate) in keeping with the factor structure of the original PCS (rumination, helplessness/hopelessness, magnification). An independent random sample of 500 responses was extracted from the larger database for these analyses. Model fit (with acceptable thresholds) was explored through the comparative fit index (CFI, > 0.95) [25], the Tucker Lewis Index (TLI, > 0.95) [25] and root mean square error of approximation (RMSEA, < 0.07) [26]. Modification indices were used as necessary to identify any potential modifications for improving fit (e.g. correlated error terms), always with the goal of a single-factor scale.

### Concurrent validity

Following the triangulation procedures (conceptual, Rasch, CFA), the remaining items were considered a short-PCS and evaluated against the original 13-item version for comparison of scale function. A sample of *n* = 400 responses was deemed adequate for estimating correlations with narrow confidence limits for comparison purposes. Bootstrapped Pearson’s r correlations with 95% confidence intervals were calculated for the association between the original and shortened version(s) with a minimum correlation of *r* = 0.90 considered acceptable agreement between the two versions. Associations with secondary variables were calculated for the Beck Depression Inventory – II, and the Physical and Affective Interference subscales of the Brief Pain Inventory. Correlation estimates that were within the 95% confidence interval of the original were considered statistically similar, lending support to construct validity.

ITCs and correlations were conducted using SPSS v22 (IBM Inc., Chicago USA).

## Results

### Conceptual review

On conceptual review of all PCS items, four were flagged due to potentially difficult or ambiguous wording. PCS item 1 ‘*I worry all the time about whether the pain will end*’ was flagged as *all the time* is also a response option, effectively leading to a potentially ambiguous response of “I worry all the time about whether the pain will end all the time”. PCS item 2 ‘*I feel I can’t go on’* was also flagged on both conceptual and safety grounds. While this item may be informed by the latent construct of catastrophizing (if interpreted as “I feel I can’t go on with my pain this severe”), it is also potentially informed by depression and suicidal ideation. Our anecdotal experience indicates that clinicians rarely consider this potential when administering and interpreting the scale, leaving the clinician vulnerable to documented evidence of possibly undiagnosed depression or suicidality. Two items were flagged as double-barreled (items 3 and 4) that could potentially lead to ambiguity where, for example, a respondent may describe the experience as terrible but not necessarily feel that it will never get any better (item 3). Item 6 ‘*I become afraid that the pain will get worse*’ was flagged as the only item in the scale that presented a future-oriented rather than current perspective. Items 5 ‘*I feel I can’t stand it any more*’ and 13 ‘*I wonder whether something serious may happen*’ were both flagged owing to the clear allusion to serious or severe problems that was arguably a poor fit to the severity-based response options; the team were unsure whether it made sense to expect a response “to a slight degree” for either of these items. These 9 items were flagged but not yet removed. The remaining items mapped adequately to the theoretical construct of ‘catastrophizing’, though some potential for redundancy was noted by the team, expected to arise in the subsequent statistical evaluations.

### Statistical review

Table 1 provides the characteristics of subjects in the overall database from which random draws were pulled for the different components of this analysis.

**Table 1:** Characteristics of the samples used for the analyses. Data are part of the Quebec Pain Registry database

	Mean (***n*** = 5646)
Age (yrs)	52.9 (16.2)
Sex (% males)	40.6
Average Pain Intensity	7.6 (1.8)
Duration (yrs)	6.9 (8.6)
Cause	
Trauma	33.5
Non-trauma	36.7
Other	29.8
Medical Legal Status	
None	80.4
Auto Insurance	4.1
Worker’s Compensation	11.0
Lawyer	4.6
Educational Attainment	
None	0.5%
Elementary School	8.4%
Secondary School	38.0%
College / Technical School	27.5%
University	25.4%

### Corrected item-total correlations

Table 2 shows the 3 corrected item-total correlation matrices constructed from 3 independent random samples of *n* = 130 each. Using our algorithm of requiring a minimum corrected item-total correlation across the 3 independent samples of *r* = 0.70 for each item, and in consideration of the prior conceptual review step, items 1 (‘I worry all the time about whether the pain will end’), 2 (‘I feel I can’t go on’), 7 (‘I keep thinking of other painful events’), 8 (‘I anxiously want the pain to go away’), 12 (‘There’s nothing I can do to reduce the intensity of the pain’) and 13 (‘I wonder whether something serious may happen’) were removed at this stage. Items 3 and 4, flagged during the conceptual review, were retained due to high ITCs. The remaining 7 items were carried forward for Rasch analysis.

**Table 2:** Corrected item-total and squared multiple correlations, evaluating each individual item against the total score of the remaining 12 items, in 3 independent samples of 130 subjects (10 subjects per item).

	Corrected item-total correlation (*N* = 130 each)		
	Sample 1	Sample 2	Sample 3
1. *I worry all the time about whether the pain will end*	*0.70 (0.60)*	*0.58 (0.50)*	*0.75 (0.59)*
2. I feel I can’t go on	0.74 (0.62)	0.66 (0.57)	0.73 (0.61)
3. **It’s terrible and I think it’s never going to get any better**	0.73 (0.67)	0.72 (0.69)	0.82 (0.74)
4. **It’s awful and I feel that it overwhelms me**	0.84 (0.76)	0.80 (0.77)	0.81 (0.75)
5. **I feel I can’t stand it anymore**	0.79 (0.71)	0.69 (0.62)	0.79 (0.69)
6. **I become afraid that the pain will get worse**	0.73 (0.60)	0.54 (0.36)	0.78 (0.67)
7. *I keep thinking of other painful events*	*0.55 (0.49)*	*0.48 (0.34)*	*0.54 (0.40)*
8. *I anxiously want the pain to go away*	*0.64 (0.58)*	*0.65 (0.49)*	*0.68 (0.59)*
9. **I can’t seem to keep it out of my mind**	0.64 (0.58)	0.66 (0.58)	0.83 (0.77)
10. **I keep thinking about how much it hurts**	0.70 (0.71)	0.69 (0.61)	0.83 (0.78)
11. **I keep thinking about how badly I want the pain to stop**	0.76 (0.75)	0.72 (0.61)	0.79 (0.72)
12. *There’s nothing I can do to reduce the intensity of the pain*	*0.60 (0.48)*	*0.52 (0.33)*	*0.68 (0.51)*
13. *I wonder whether something serious may happen*	*0.64 (0.58)*	*0.55 (0.39)*	*0.69 (0.54)*

All values are Pearson’s r. *Italics*: Items that did not reach the a priori threshold for retention of mean item-total correlation at least *r* = 0.70. Bolded: Items that were retained to move onto Rasch analysis on the basis of either statistical or conceptual considerations

### Rasch analysis

**Fig. 1:**
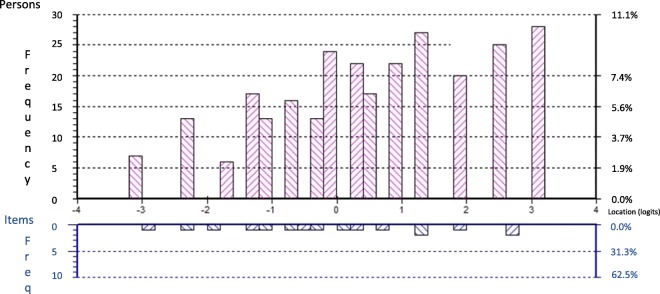
Person-Item Threshold histogram from Rasch analysis using the first random sample of *n* = 250, showing good coverage of the person locations by the thresholds of the brief scale

Partial credit Rasch modeling was conducted using the first random sample of 250 subjects. The 7-item model showed significant misfit to the Rasch model (χ^2^ = 42.65, *p* = 0.003). Item 6 had a fit residual of 2.93 logits, while no other item showed a residual of > 1.29 logits. Removal of item 6 (‘I become afraid that the pain will get worse’), also flagged in the conceptual review, led to acceptable fit (χ^2^ = 21.58, *p* = 0.25). Items 5 and 10 then revealed significant DIF by sex, and consistent with the conceptual review, item 5 (‘I feel I can’t stand it anymore’) had a single disordered response threshold where option 1 was never more likely to be chosen than the remaining options. Removal of item 5 led to significantly improved fit again (χ^2^ = 14.38, *p* = 0.50, PSI = 0.83), and resolved the DIF of item 10. Paired t-test revealed adequate unidimensionality. Repeating the analysis with a second randomly-drawn independent sample led to nearly identical fit results (χ^2^ = 17.30, *p* = 0.30). The histogram in Fig. 1 shows the logit-transformed person and item location distributions. The remaining 5 items (3, 4, 9, 10, 11) were moved onto CFA.

### Confirmatory factor analysis

**Fig. 2:**
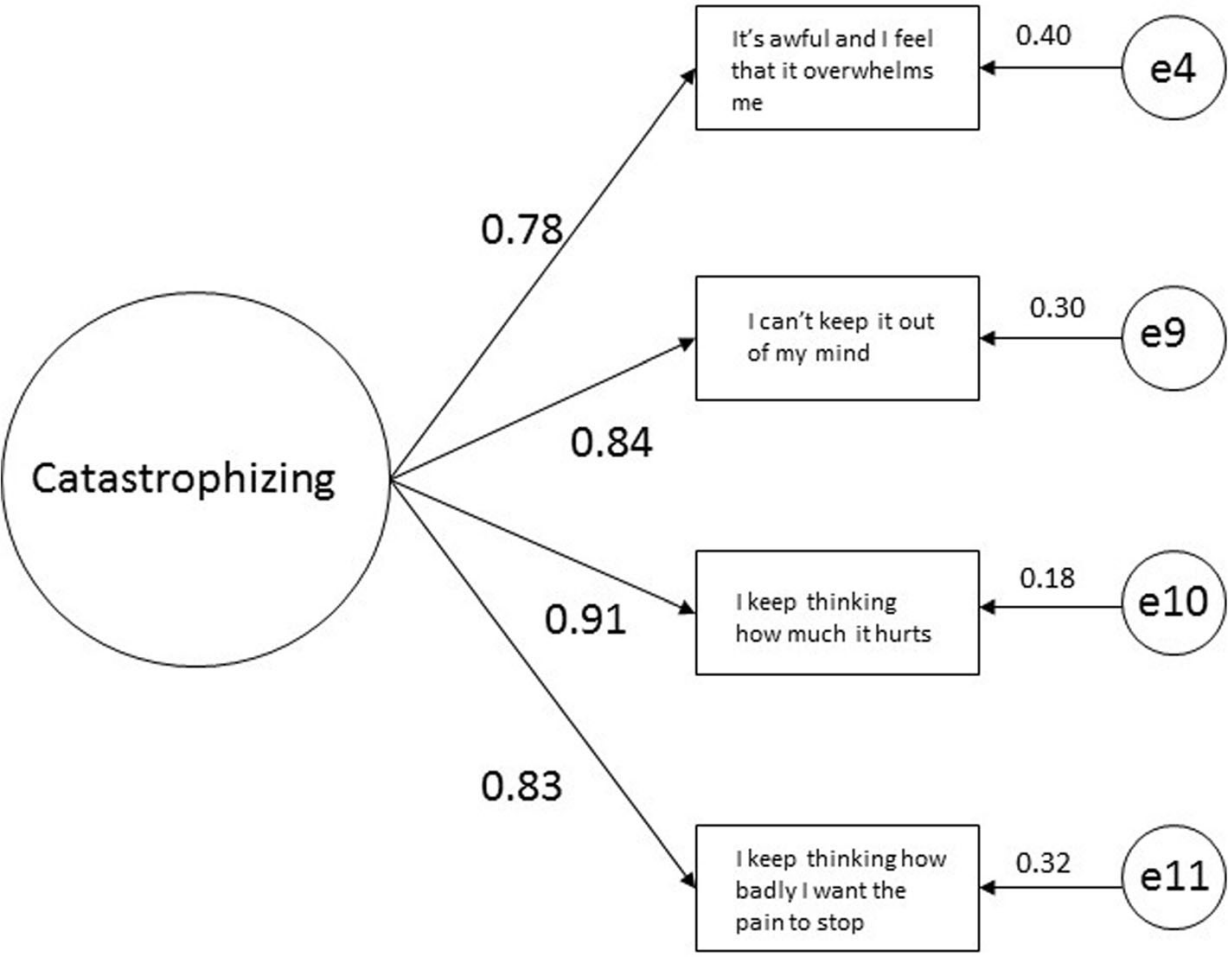
Unrestricted, standardized path coefficients through confirmatory factor analysis on *n* = 500 scores. e = error (residual) term

In another independent random sample of 500 (*n* = 494 after removal of missing responses), despite evidence of unidimensionality in Rasch modeling, CFA fit indicators revealed unacceptable fit by virtue of high RMSEA when the 5 remaining items were loaded on a single latent factor (CFI = 0.99, TLI = 0.98, RMSEA = 0.19). Model fit reached acceptable thresholds when either a 2-factor (Hopelessness/Helplessness and Rumination) model was tested (CFI = 1.00, TLI = 1.00, RMSEA = 0.05) or when the item with the largest residual (item 3) was removed leaving items 4, 9, 10, 11 (χ^2^= 3.33 *p*=0.19, CFI = 1.00, TLI = 1.00, RMSEA = 0.05). As item 3 was also flagged in the conceptual analysis and because of the risk of over-fitting the CFA model by virtue of perfect CFI and TLI, we returned to Rasch modeling to explore model fit with that item removed. Fit to the Rasch model remained acceptable (χ^2^ = 20.26, *p* = 0.06). With only 4 items the Rasch-based PSI suffered (PSI from 0.83 to 0.76) but remained adequately reliable for group-level use [23]. Revisiting the overall purpose of creating a shortened scale that could be statistically interpreted as a single summative score, the 4-item version satisfied that purpose from newer (Rasch-based) and Classical (CFA-based) perspectives and was in accordance with the conceptual review. Fig. 2 shows the factor loadings of the 4 retained items. The 4-item version was therefore retained for concurrent analyses.

### Concurrent validity between original and new brief versions

Table 3 shows the bivariate correlations between the full (13-item) and shortened (4-item) versions of the PCS using using an independent random drawing of *n* = 400 responses. The correlation between the versions was nearly perfect for the sample (*r* = 0.94). Random replacement bootstrapped correlations with 95% confidence intervals revealed identical associations between the short and full PCS versions and each of external metrics: BDI-II (*r* = 0.50 full, 0.46 short), BPI Physical Interference (*r* = 0.47 full, 0.43 short) and BPI Affective Interference (*r* = 0.58 full, 0.56 short). In all cases point estimates for Pearson’s r were slightly lower for the short version compared to the full, but in no case were the differences greater than *r* = 0.04 points and none were statistically different by virtue of overlapping 95% confidence intervals.

**Table 3:** Bootstrapped pearson’s correlation estimates (r) based on a sample of 400 responses (*n* = 378 after removal of those with missing data)

	Chronic	
	PCS full	BriefPCS-chronic
PCS Full	N/A	0.94** (0.93, 0.96)
BDI-II	0.50** (0.44, 0.57)	0.46** (0.39, 0.53)
BPI Physical	0.47** (0.38, 0.55)	0.43** (0.39, 0.53)
BPI Affective	0.58** (0.51, 0.64)	0.56** (0.49, 0.62)

**: correlation significant at the *p* < 0.01 level

## Discussion

The Pain Catastrophizing Scale has become a popular self-report tool in pain-related research. It is often used as a screening tool to discriminate between people high and low in catastrophic beliefs rather than as an evaluative measure to track change over time. While scales with more items are generally thought to be more responsive to change by virtue of a larger range of scores, discriminative/screening tools can function adequately well with fewer items. While there is no ‘right’ number of items required for a screening tool, scales of 3 to 5 items have been shown to have adequate discriminative properties for several clinical conditions [27, 28]. If the intention of the PCS is to discriminate between high/low catastrophizers for patient phenotyping, then the smallest number of items that retain adequate measurement properties should reduce barriers to implementation in practice. Further, while the original PCS was described as a 3-factor scale, it is rare that the 3 separate subscale scores are reported in pain research, and even rarer still that those scores can or have been used to inform different treatment directions. With these pragmatic considerations in mind, our team set out to intentionally reduce the number of PCS items as far as possible while retaining sound measurement properties for screening purposes that also retained strong association with the full original scale.

The approach was rigorous, harnessing knowledge and methods from qualitative and quantitative fields of psychometrics and scale development. No single piece of evidence was used in isolation to decide on item retention/removal. As a deviation from traditional approaches that tend to heavily prioritize statistical methods, we have demonstrated an approach to triangulating findings across techniques to inform revision decisions. The size of the QPR database provided the opportunity to test and retest the scales as needed. Through conscientious, informed decisions a new *brief* version of the scale was created that satisfied theory, classical, and newer statistical methods in a way that prior attempts to shorten the scale have not done.

The results of the CFA revealed some ambiguity in model fit; despite unidimensionality in Rasch analysis, RMSEA was higher than desirable in the 5-item model. The goals of this study continued to guide decision making, and so one item was removed that, upon retesting in Rasch and CFA, fit both the newer and classical approaches and satisfied the conceptual review. Through this iterative and stepwise approach of testing across multiple perspectives, revising and retesting where necessary using several independent samples, we are confident that the BriefPCS version defined here is adequate for routine use. The strong correlations between the brief version and the full-scale analog (*r* ≥ 0.94) suggest that the brief version is also an adequate proxy of the full version. With only 4 items, ability to detect change over time at the individual level has likely suffered (as evidenced partly by a PSI < 0.80 in the sample), but as a screening/discriminative tool the 4-item version still offers 17 levels of discrimination (0–16). While not formally tested in this study, it should be expected that the score thresholds often referred to in the existing PCS literature (20/52 moderate, 30/52 high catastrophizing) can safely be applied to the new scale (6/16 moderate, 9/16 high) though this is a reasonable area for additional study.

The brief scale offers additional benefits. Perhaps the most notable is the removal of item 2 ‘*I feel I can’t go on*’ that was deemed through conceptual analysis to be potentially tapping depression or even suicidal ideation. While these are important constructs that should be explored especially in especially in people with chronic pain, anecdotal experience with clinicians using this scale reveal that few are aware of this potential overlap, and fewer still act upon it when endorsed. While this may be a trivial concern, the team unanimously decided to remove it from the brief version due partly to statistical considerations but also in the interest of protecting clinicians who may be ill-equipped to address emotional crises, and to highlight that depression/suicidality in chronic pain warrants its own dedicated investigation. The removal of items 8 and 12 also fit with results from our prior Rasch analysis of the PCS in an independent sample [13]. That analysis found disordered response thresholds requiring rescoring of those two items, and evidence of considerable location dependence between other items through the scale. Collectively those prior findings lent additional justification to the effort to create an abbreviated scale that overcomes some challenges to scoring and interpretation in the original version.

Prior attempts at abbreviating the PCS have prioritized the retention of a 3-factor structure. Bot [18] and colleagues derived a 4-item version of the tool that retained the 3 factors but suffered lower correlation with the full 13-item version than the version derived here, with Pearson r values ranging from *r* = 0.60 to 0.82. McWilliams and colleagues [16] derived a 6-item version that also prioritized the inclusion of items from all 3 subscales. That version retained items 4, 5, 6, 10, 11 and 13, three of which (4, 10 and 11) overlapped with our version, and correlated with the original version at a similar magnitude (*r* = 0.95). Further in our larger sample, correlations between the PCS-4 and depression (*r* = 0.46) and functional interference (*r* = 0.43) were similar in absolute magnitude to those of McWilliams’ 6-item version (*r* = 0.47 depression, and 0.38 functional interference). We believe the rigour with which our 4-item version was derived and its brevity represents an advantage over the 6-item version of McWilliams, though the potential ability to tap the 3 different subscales of the original PCS in the McWilliams version may be an attractive aspect for those who find value in doing so. Darnall and colleagues [17] 3-item version also prioritized inclusion of items from all 3 subscales. That version retained items 4, 6, and 10, two of which (4 and 10) were again consistent with the version described herein. Those authors did not analyze correlation with the original version making comparison difficult. That group also made other changes to the scale instructions and scoring to make it appropriate as a tool for daily ‘state’ administration rather than as a clinical phenotyping tool of catastrophizing as a ‘trait’. The availability of these different versions has value, and users are encouraged to consider the intended use of a scale before choosing the abbreviated version that will best fit their needs.

Despite considerable rigor there are limitations that should be observed when interpreting these results. The primary one is that this was a secondary review of an existing database that meant our research team had no control over the methods through which the primary data were collected. While this is less concerning with patient self-report data than it would have been if using clinician-administered tools, it does mean that logistics such as instructions to patients, time allowed to complete, and even environment within which the tools were completed very likely differed across sampling context. The extent to which this may have influenced results is unknown, and this is one reason for analysis on multiple random samples. Another potential influence on our results is that fit indices for the CFA may have been artificially biased as no covariates were built into the model. By that point, covariates had already been explored through the DIF analyses in Rasch. However, it is possible that the CFA fit indicators could have been improved even further by inclusion of, for example, sex or age in the CFA analysis. With CFI/TLI estimates already at or near 1.00, it is doubtful that adding complexity to an already complex analysis would have led to any meaningful change in the results or interpretation, and we were already concerned about creating an unstable model through overfitting. Finally, as with any scale revision, confidence in the results will be strengthened when the new version is tested again in another independent sample and results are replicated.

## Conclusion

This study has described a rigorous approach to scale item reduction through triangulation across statistical and qualitative perspectives to arrive at what appears to be a psychometrically and theoretically sound, unidimensional, brief version of the PCS for use in people with chronic pain. The Brief PCS is easy to score, strongly associated with the full original version, and equally associated with other secondary outcomes. Where deeper exploration of pain related catastrophizing (and its sub-factors) is required, the full version should still be used. Where quick identification of those low or high in catastrophic beliefs is the desired outcome, this new brief version should suffice.

## Summary statement

A new brief version of the Pain Catastrophizing Scale has been derived that shows sound measurement properties and strong association with the full 13-item original.

## Data Availability

The data for this study were used under permission and through a signed data transfer agreement with the Quebec Pain Registry. Those wishing to access the data can do so, though limitations and licensing agreements exist, through www.quebecpainregistry.com.

## Abbreviations

PCS: Pain Catastrophizing Scale
QPR: Quebec Pain Registry
CFI: Cumulative Fit Index
TLI: Tucker Lewis Index
RMSEA: Root Mean Square Error of Approximation
ITC: Item-Total Correlation
DIF: Differential Item Functioning
PSI: Person Separation Index
BDI-II: Beck Depression Inventory version 2
BPI: Brief Pain Inventory
